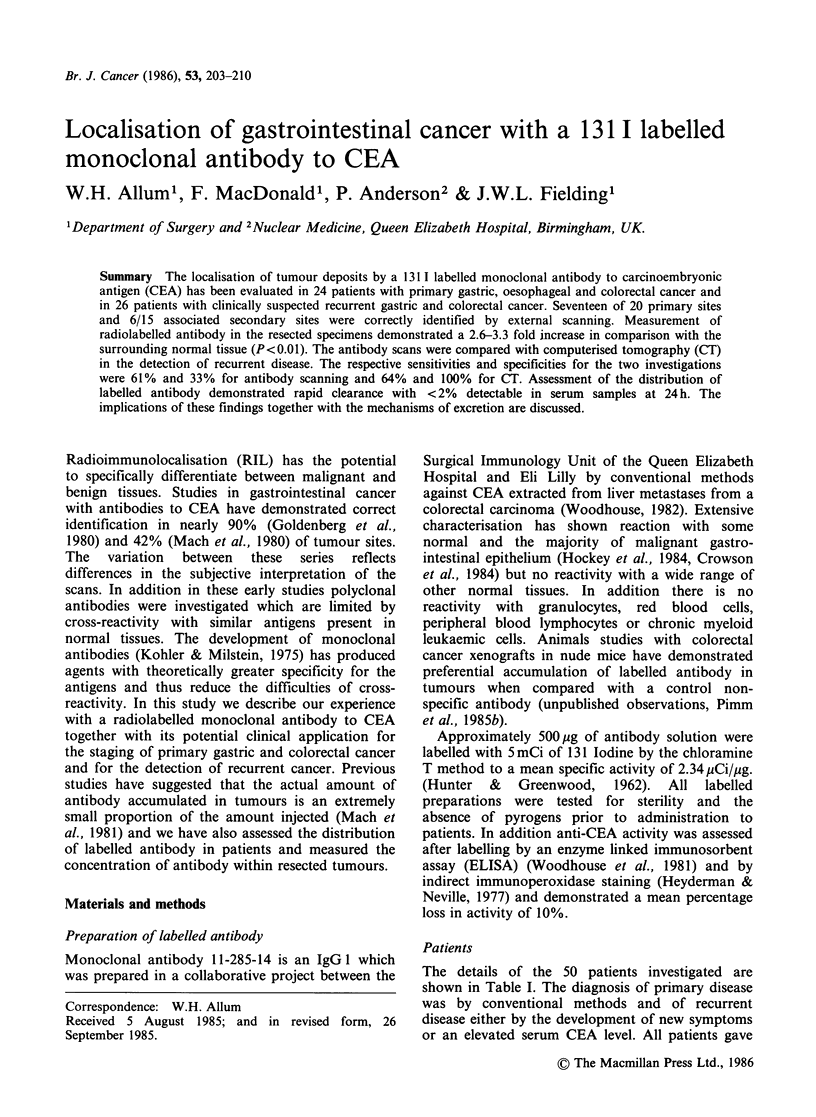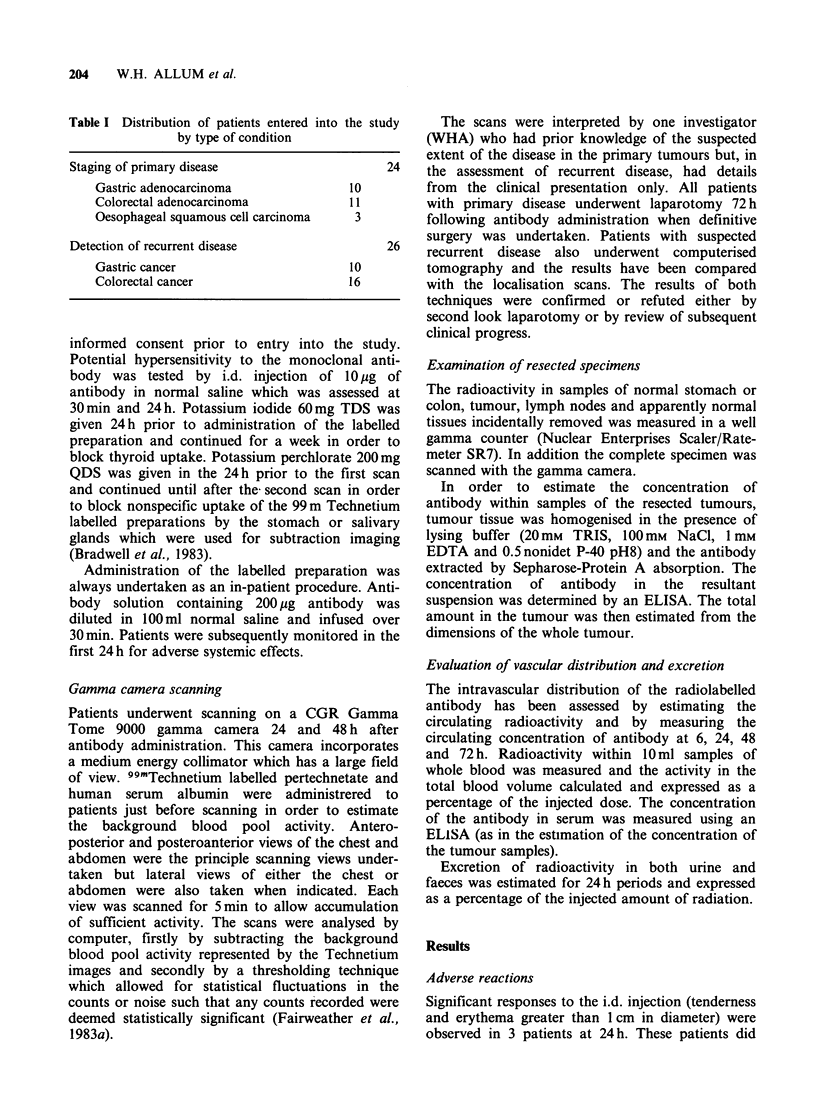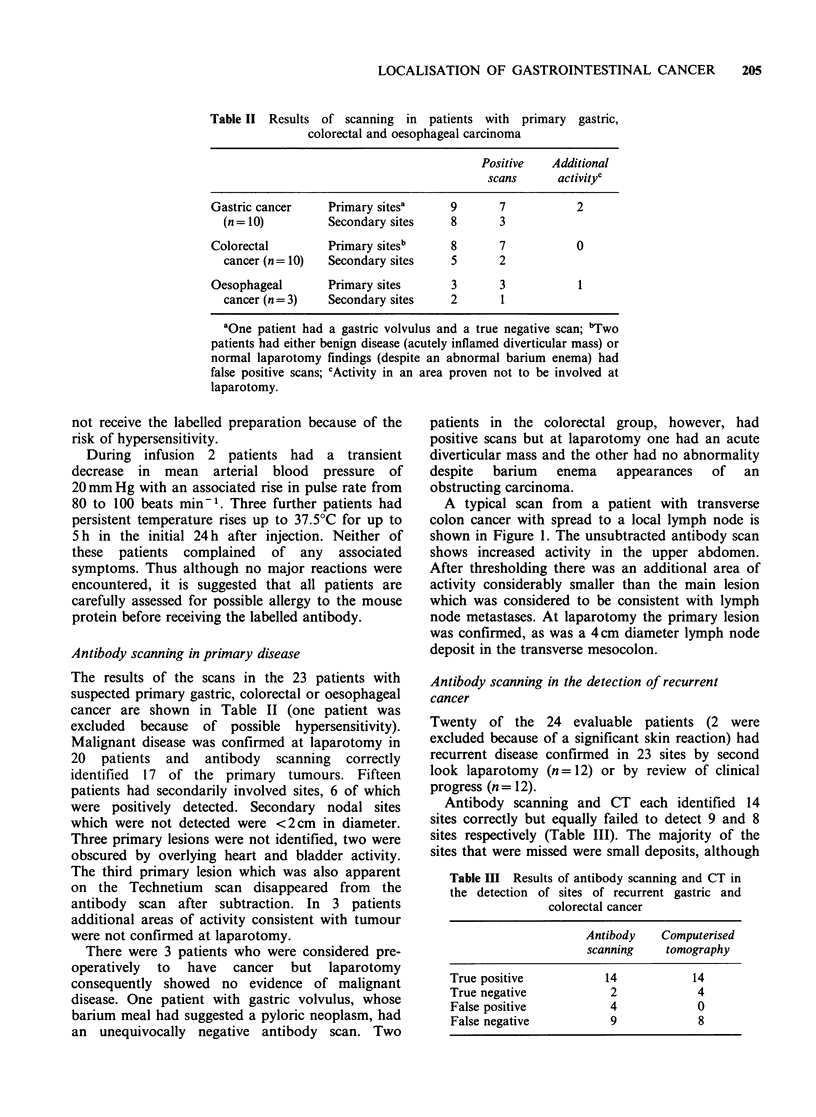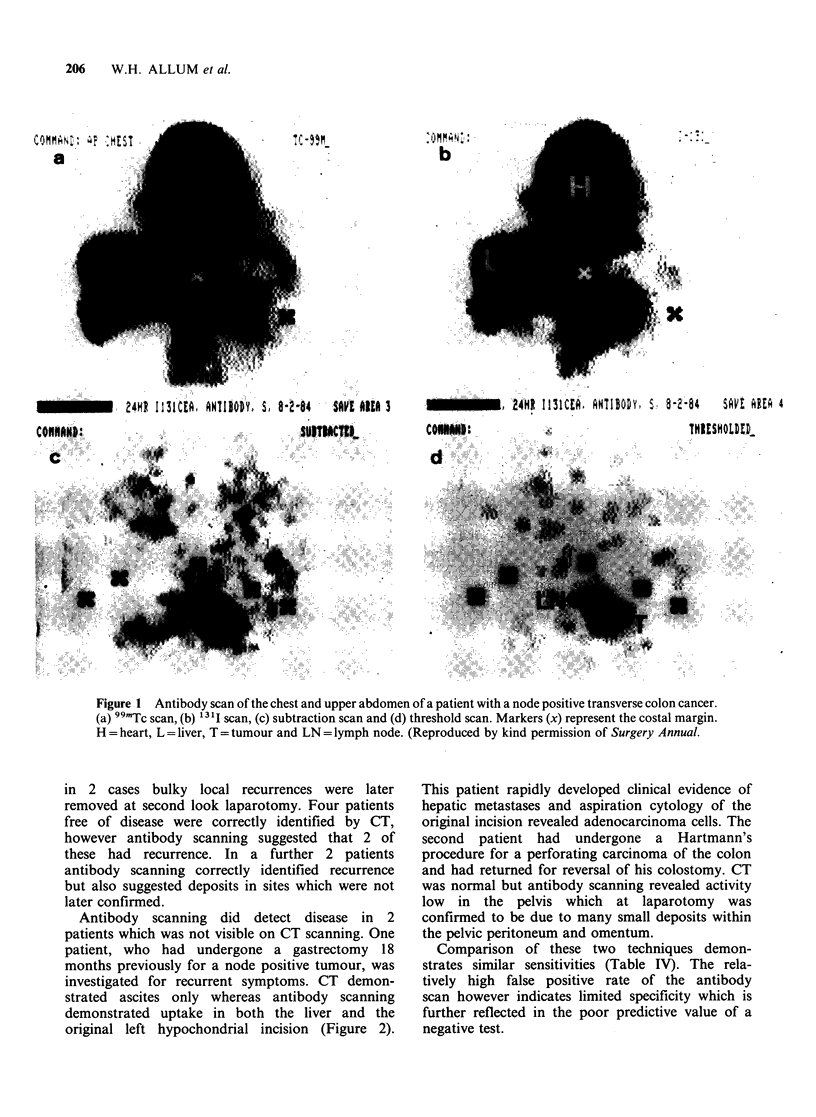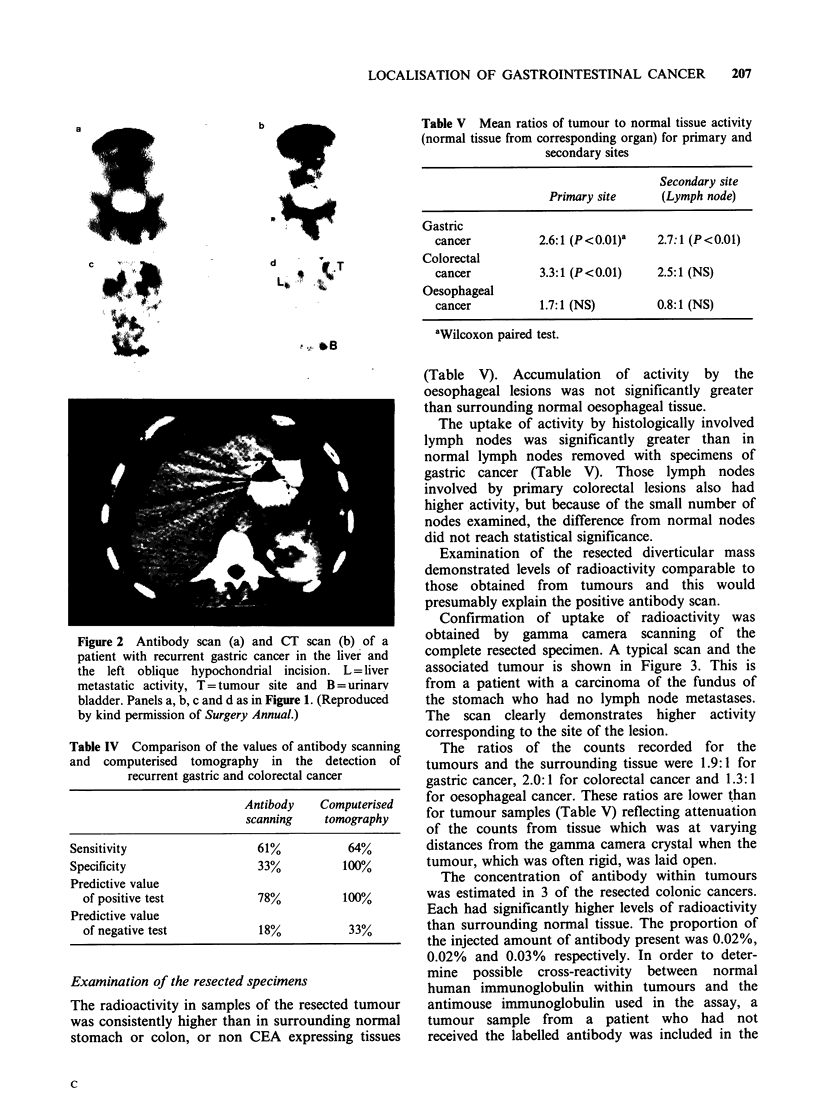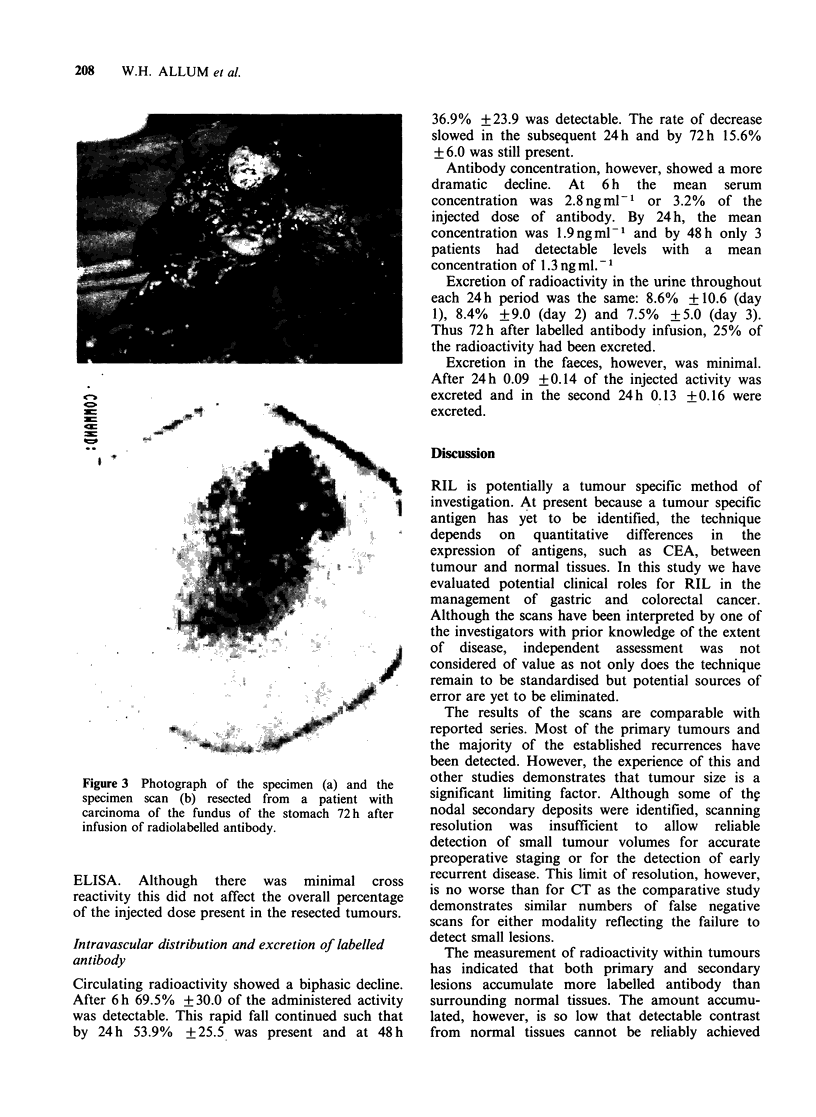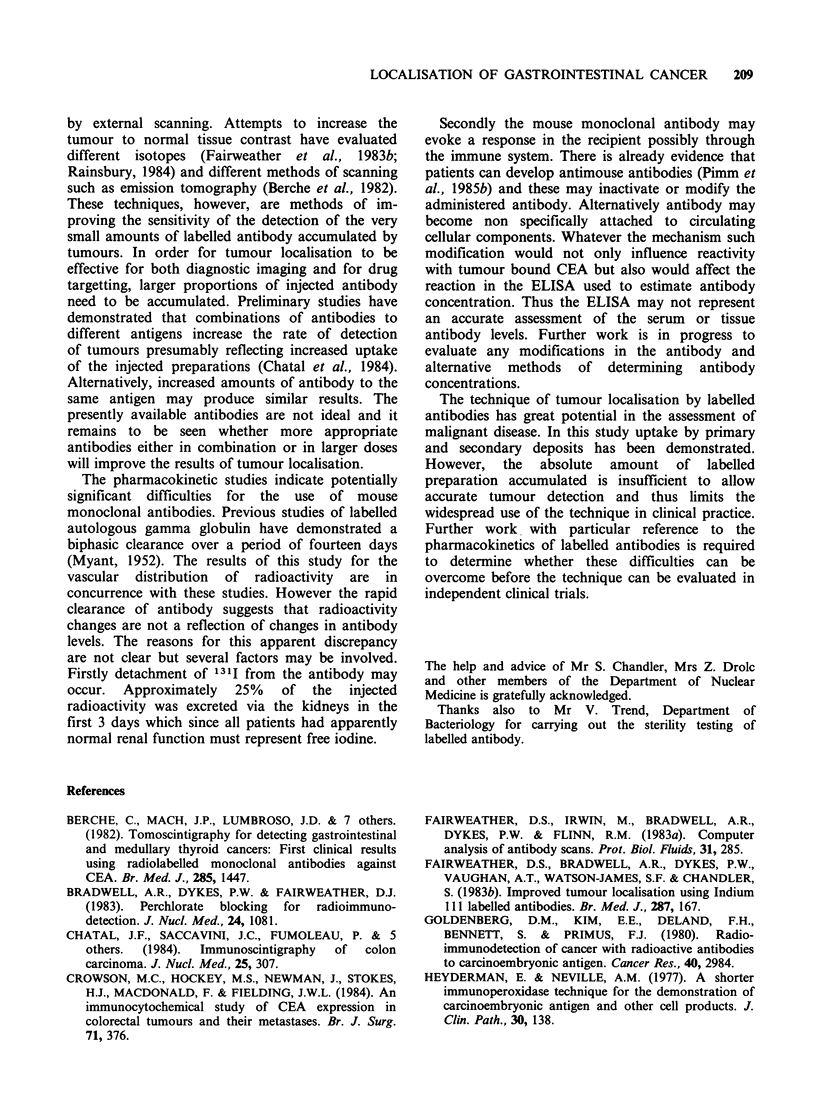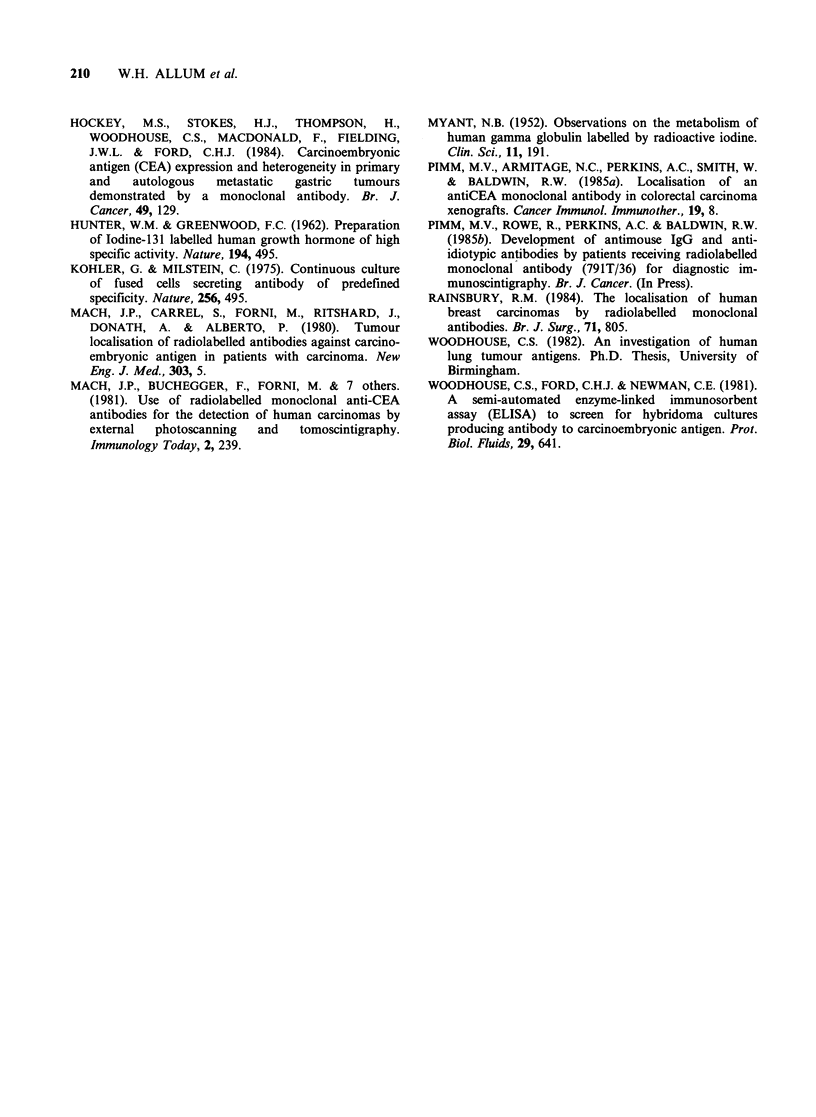# Localisation of gastrointestinal cancer with a 131 I labelled monoclonal antibody to CEA.

**DOI:** 10.1038/bjc.1986.36

**Published:** 1986-02

**Authors:** W. H. Allum, F. MacDonald, P. Anderson, J. W. Fielding

## Abstract

**Images:**


*Br. J. Cancer* (1986), **53**, 203–210

### Localisation of gastrointestinal cancer with a 131 I labelled monoclonal antibody to CEA

W.H. Allum[1], F. MacDonald[1], P. Anderson[2] & J.W.L. Fielding[1]

[1]*Department of Surgery and* [2]*Nuclear Medicine, Queen Elizabeth Hospital, Birmingham, UK.*

**Summary** The localisation of tumour deposits by a 131 I labelled monoclonal antibody to carcinoembryonic antigen (CEA) has been evaluated in 24 patients with primary gastric, oesophageal and colorectal cancer and in 26 patients with clinically suspected recurrent gastric and colorectal cancer. Seventeen of 20 primary sites and 6/15 associated secondary sites were correctly identified by external scanning. Measurement of radiolabelled antibody in the resected specimens demonstrated a 2.6–3.3 fold increase in comparison with the surrounding normal tissue ($P<0.01$). The antibody scans were compared with computerised tomography (CT) in the detection of recurrent disease. The respective sensitivities and specificities for the two investigations were 61% and 33% for antibody scanning and 64% and 100% for CT. Assessment of the distribution of labelled antibody demonstrated rapid clearance with <2% detectable in serum samples at 24 h. The implications of these findings together with the mechanisms of excretion are discussed.

Radioimmunolocalisation (RIL) has the potential to specifically differentiate between malignant and benign tissues. Studies in gastrointestinal cancer with antibodies to CEA have demonstrated correct identification in nearly 90% (Goldenberg *et al.*, 1980) and 42% (Mach *et al.*, 1980) of tumour sites. The variation between these series reflects differences in the subjective interpretation of the scans. In addition in these early studies polyclonal antibodies were investigated which are limited by cross-reactivity with similar antigens present in normal tissues. The development of monoclonal antibodies (Kohler & Milstein, 1975) has produced agents with theoretically greater specificity for the antigens and thus reduce the difficulties of cross-reactivity. In this study we describe our experience with a radiolabelled monoclonal antibody to CEA together with its potential clinical application for the staging of primary gastric and colorectal cancer and for the detection of recurrent cancer. Previous studies have suggested that the actual amount of antibody accumulated in tumours is an extremely small proportion of the amount injected (Mach *et al.*, 1981) and we have also assessed the distribution of labelled antibody in patients and measured the concentration of antibody within resected tumours.

### Materials and methods

### *Preparation of labelled antibody*

Monoclonal antibody 11-285-14 is an IgG 1 which was prepared in a collaborative project between the Surgical Immunology Unit of the Queen Elizabeth Hospital and Eli Lilly by conventional methods against CEA extracted from liver metastases from a colorectal carcinoma (Woodhouse, 1982). Extensive characterisation has shown reaction with some normal and the majority of malignant gastrointestinal epithelium (Hockey *et al.*, 1984, Crowson *et al.*, 1984) but no reactivity with a wide range of other normal tissues. In addition there is no reactivity with granulocytes, red blood cells, peripheral blood lymphocytes or chronic myeloid leukaemic cells. Animals studies with colorectal cancer xenografts in nude mice have demonstrated preferential accumulation of labelled antibody in tumours when compared with a control non-specific antibody (unpublished observations, Pimm *et al.*, 1985*b*).

Approximately 500 $\mu$g of antibody solution were labelled with 5 mCi of 131 Iodine by the chloramine T method to a mean specific activity of 2.34 $\mu$Ci/$\mu$g. (Hunter & Greenwood, 1962). All labelled preparations were tested for sterility and the absence of pyrogens prior to administration to patients. In addition anti-CEA activity was assessed after labelling by an enzyme linked immunosorbent assay (ELISA) (Woodhouse *et al.*, 1981) and by indirect immunoperoxidase staining (Heyderman & Neville, 1977) and demonstrated a mean percentage loss in activity of 10%.

### *Patients*

The details of the 50 patients investigated are shown in Table I. The diagnosis of primary disease was by conventional methods and of recurrent disease either by the development of new symptoms or an elevated serum CEA level. All patients gave

Correspondence: W.H. Allum
Received 5 August 1985; and in revised form, 26 September 1985.

© The Macmillan Press Ltd., 1986

Table I Distribution of patients entered into the study by type of condition

| | | |
|---|---|---|
| Staging of primary disease | | 24 |
| Gastric adenocarcinoma | 10 | |
| Colorectal adenocarcinoma | 11 | |
| Oesophageal squamous cell carcinoma | 3 | |
| Detection of recurrent disease | | 26 |
| Gastric cancer | 10 | |
| Colorectal cancer | 16 | |

informed consent prior to entry into the study. Potential hypersensitivity to the monoclonal antibody was tested by i.d. injection of 10 μg of antibody in normal saline which was assessed at 30 min and 24 h. Potassium iodide 60 mg TDS was given 24 h prior to administration of the labelled preparation and continued for a week in order to block thyroid uptake. Potassium perchlorate 200 mg QDS was given in the 24 h prior to the first scan and continued until after the second scan in order to block nonspecific uptake of the 99 m Technetium labelled preparations by the stomach or salivary glands which were used for subtraction imaging (Bradwell *et al.*, 1983).

Administration of the labelled preparation was always undertaken as an in-patient procedure. Antibody solution containing 200 μg antibody was diluted in 100 ml normal saline and infused over 30 min. Patients were subsequently monitored in the first 24 h for adverse systemic effects.

*Gamma camera scanning*

Patients underwent scanning on a CGR Gamma Tome 9000 gamma camera 24 and 48 h after antibody administration. This camera incorporates a medium energy collimator which has a large field of view. $^{99m}$Technetium labelled pertechnetate and human serum albumin were administrered to patients just before scanning in order to estimate the background blood pool activity. Anteroposterior and posteroanterior views of the chest and abdomen were the principle scanning views undertaken but lateral views of either the chest or abdomen were also taken when indicated. Each view was scanned for 5 min to allow accumulation of sufficient activity. The scans were analysed by computer, firstly by subtracting the background blood pool activity represented by the Technetium images and secondly by a thresholding technique which allowed for statistical fluctuations in the counts or noise such that any counts recorded were deemed statistically significant (Fairweather *et al.*, 1983*a*).

The scans were interpreted by one investigator (WHA) who had prior knowledge of the suspected extent of the disease in the primary tumours but, in the assessment of recurrent disease, had details from the clinical presentation only. All patients with primary disease underwent laparotomy 72 h following antibody administration when definitive surgery was undertaken. Patients with suspected recurrent disease also underwent computerised tomography and the results have been compared with the localisation scans. The results of both techniques were confirmed or refuted either by second look laparotomy or by review of subsequent clinical progress.

*Examination of resected specimens*

The radioactivity in samples of normal stomach or colon, tumour, lymph nodes and apparently normal tissues incidentally removed was measured in a well gamma counter (Nuclear Enterprises Scaler/Ratemeter SR7). In addition the complete specimen was scanned with the gamma camera.

In order to estimate the concentration of antibody within samples of the resected tumours, tumour tissue was homogenised in the presence of lysing buffer (20 mM TRIS, 100 mM NaCl, 1 mM EDTA and 0.5 nonidet P-40 pH8) and the antibody extracted by Sepharose-Protein A absorption. The concentration of antibody in the resultant suspension was determined by an ELISA. The total amount in the tumour was then estimated from the dimensions of the whole tumour.

*Evaluation of vascular distribution and excretion*

The intravascular distribution of the radiolabelled antibody has been assessed by estimating the circulating radioactivity and by measuring the circulating concentration of antibody at 6, 24, 48 and 72 h. Radioactivity within 10 ml samples of whole blood was measured and the activity in the total blood volume calculated and expressed as a percentage of the injected dose. The concentration of the antibody in serum was measured using an ELISA (as in the estimation of the concentration of the tumour samples).

Excretion of radioactivity in both urine and faeces was estimated for 24 h periods and expressed as a percentage of the injected amount of radiation.

### Results

*Adverse reactions*

Significant responses to the i.d. injection (tenderness and erythema greater than 1 cm in diameter) were observed in 3 patients at 24 h. These patients did

**Table II** Results of scanning in patients with primary gastric, colorectal and oesophageal carcinoma

| | | | *Positive scans* | *Additional activity*[c] |
|---|---|---|---|---|
| Gastric cancer ($n=10$) | Primary sites[a] | 9 | 7 | 2 |
| | Secondary sites | 8 | 3 | |
| Colorectal cancer ($n=10$) | Primary sites[b] | 8 | 7 | 0 |
| | Secondary sites | 5 | 2 | |
| Oesophageal cancer ($n=3$) | Primary sites | 3 | 3 | 1 |
| | Secondary sites | 2 | 1 | |

[a]One patient had a gastric volvulus and a true negative scan; [b]Two patients had either benign disease (acutely inflamed diverticular mass) or normal laparotomy findings (despite an abnormal barium enema) had false positive scans; [c]Activity in an area proven not to be involved at laparotomy.

not receive the labelled preparation because of the risk of hypersensitivity.

During infusion 2 patients had a transient decrease in mean arterial blood pressure of 20 mm Hg with an associated rise in pulse rate from 80 to 100 beats $min^{-1}$. Three further patients had persistent temperature rises up to 37.5°C for up to 5 h in the initial 24 h after injection. Neither of these patients complained of any associated symptoms. Thus although no major reactions were encountered, it is suggested that all patients are carefully assessed for possible allergy to the mouse protein before receiving the labelled antibody.

*Antibody scanning in primary disease*

The results of the scans in the 23 patients with suspected primary gastric, colorectal or oesophageal cancer are shown in Table II (one patient was excluded because of possible hypersensitivity). Malignant disease was confirmed at laparotomy in 20 patients and antibody scanning correctly identified 17 of the primary tumours. Fifteen patients had secondarily involved sites, 6 of which were positively detected. Secondary nodal sites which were not detected were $<2$ cm in diameter. Three primary lesions were not identified, two were obscured by overlying heart and bladder activity. The third primary lesion which was also apparent on the Technetium scan disappeared from the antibody scan after subtraction. In 3 patients additional areas of activity consistent with tumour were not confirmed at laparotomy.

There were 3 patients who were considered pre-operatively to have cancer but laparotomy consequently showed no evidence of malignant disease. One patient with gastric volvulus, whose barium meal had suggested a pyloric neoplasm, had an unequivocally negative antibody scan. Two patients in the colorectal group, however, had positive scans but at laparotomy one had an acute diverticular mass and the other had no abnormality despite barium enema appearances of an obstructing carcinoma.

A typical scan from a patient with transverse colon cancer with spread to a local lymph node is shown in Figure 1. The unsubtracted antibody scan shows increased activity in the upper abdomen. After thresholding there was an additional area of activity considerably smaller than the main lesion which was considered to be consistent with lymph node metastases. At laparotomy the primary lesion was confirmed, as was a 4 cm diameter lymph node deposit in the transverse mesocolon.

*Antibody scanning in the detection of recurrent cancer*

Twenty of the 24 evaluable patients (2 were excluded because of a significant skin reaction) had recurrent disease confirmed in 23 sites by second look laparotomy ($n=12$) or by review of clinical progress ($n=12$).

Antibody scanning and CT each identified 14 sites correctly but equally failed to detect 9 and 8 sites respectively (Table III). The majority of the sites that were missed were small deposits, although

**Table III** Results of antibody scanning and CT in the detection of sites of recurrent gastric and colorectal cancer

| | *Antibody scanning* | *Computerised tomography* |
|---|---|---|
| True positive | 14 | 14 |
| True negative | 2 | 4 |
| False positive | 4 | 0 |
| False negative | 9 | 8 |

**Figure 1** Antibody scan of the chest and upper abdomen of a patient with a node positive transverse colon cancer. (a) $^{99m}$Tc scan, (b) $^{131}$I scan, (c) subtraction scan and (d) threshold scan. Markers ($x$) represent the costal margin. H = heart, L = liver, T = tumour and LN = lymph node. (Reproduced by kind permission of *Surgery Annual*.

in 2 cases bulky local recurrences were later removed at second look laparotomy. Four patients free of disease were correctly identified by CT, however antibody scanning suggested that 2 of these had recurrence. In a further 2 patients antibody scanning correctly identified recurrence but also suggested deposits in sites which were not later confirmed.

Antibody scanning did detect disease in 2 patients which was not visible on CT scanning. One patient, who had undergone a gastrectomy 18 months previously for a node positive tumour, was investigated for recurrent symptoms. CT demonstrated ascites only whereas antibody scanning demonstrated uptake in both the liver and the original left hypochondrial incision (Figure 2). This patient rapidly developed clinical evidence of hepatic metastases and aspiration cytology of the original incision revealed adenocarcinoma cells. The second patient had undergone a Hartmann's procedure for a perforating carcinoma of the colon and had returned for reversal of his colostomy. CT was normal but antibody scanning revealed activity low in the pelvis which at laparotomy was confirmed to be due to many small deposits within the pelvic peritoneum and omentum.

Comparison of these two techniques demonstrates similar sensitivities (Table IV). The relatively high false positive rate of the antibody scan however indicates limited specificity which is further reflected in the poor predictive value of a negative test.

**Figure 2** Antibody scan (a) and CT scan (b) of a patient with recurrent gastric cancer in the liver and the left oblique hypochondrial incision. L = liver metastatic activity, T = tumour site and B = urinary bladder. Panels a, b, c and d as in **Figure 1**. (Reproduced by kind permission of *Surgery Annual.*)

**Table IV** Comparison of the values of antibody scanning and computerised tomography in the detection of recurrent gastric and colorectal cancer

| | *Antibody scanning* | *Computerised tomography* |
|---|---|---|
| Sensitivity | 61% | 64% |
| Specificity | 33% | 100% |
| Predictive value of positive test | 78% | 100% |
| Predictive value of negative test | 18% | 33% |

*Examination of the resected specimens*

The radioactivity in samples of the resected tumour was consistently higher than in surrounding normal stomach or colon, or non CEA expressing tissues

**Table V** Mean ratios of tumour to normal tissue activity (normal tissue from corresponding organ) for primary and secondary sites

| | *Primary site* | *Secondary site (Lymph node)* |
|---|---|---|
| Gastric cancer | 2.6:1 ($P<0.01$)[a] | 2.7:1 ($P<0.01$) |
| Colorectal cancer | 3.3:1 ($P<0.01$) | 2.5:1 (NS) |
| Oesophageal cancer | 1.7:1 (NS) | 0.8:1 (NS) |

[a]Wilcoxon paired test.

(Table V). Accumulation of activity by the oesophageal lesions was not significantly greater than surrounding normal oesophageal tissue.

The uptake of activity by histologically involved lymph nodes was significantly greater than in normal lymph nodes removed with specimens of gastric cancer (Table V). Those lymph nodes involved by primary colorectal lesions also had higher activity, but because of the small number of nodes examined, the difference from normal nodes did not reach statistical significance.

Examination of the resected diverticular mass demonstrated levels of radioactivity comparable to those obtained from tumours and this would presumably explain the positive antibody scan.

Confirmation of uptake of radioactivity was obtained by gamma camera scanning of the complete resected specimen. A typical scan and the associated tumour is shown in Figure 3. This is from a patient with a carcinoma of the fundus of the stomach who had no lymph node metastases. The scan clearly demonstrates higher activity corresponding to the site of the lesion.

The ratios of the counts recorded for the tumours and the surrounding tissue were 1.9:1 for gastric cancer, 2.0:1 for colorectal cancer and 1.3:1 for oesophageal cancer. These ratios are lower than for tumour samples (Table V) reflecting attenuation of the counts from tissue which was at varying distances from the gamma camera crystal when the tumour, which was often rigid, was laid open.

The concentration of antibody within tumours was estimated in 3 of the resected colonic cancers. Each had significantly higher levels of radioactivity than surrounding normal tissue. The proportion of the injected amount of antibody present was 0.02%, 0.02% and 0.03% respectively. In order to determine possible cross-reactivity between normal human immunoglobulin within tumours and the antimouse immunoglobulin used in the assay, a tumour sample from a patient who had not received the labelled antibody was included in the

**Figure 3** Photograph of the specimen (a) and the specimen scan (b) resected from a patient with carcinoma of the fundus of the stomach 72 h after infusion of radiolabelled antibody.

ELISA. Although there was minimal cross reactivity this did not affect the overall percentage of the injected dose present in the resected tumours.

*Intravascular distribution and excretion of labelled antibody*

Circulating radioactivity showed a biphasic decline. After 6 h 69.5% $\pm$30.0 of the administered activity was detectable. This rapid fall continued such that by 24 h 53.9% $\pm$25.5 was present and at 48 h 36.9% $\pm$23.9 was detectable. The rate of decrease slowed in the subsequent 24 h and by 72 h 15.6% $\pm$6.0 was still present.

Antibody concentration, however, showed a more dramatic decline. At 6 h the mean serum concentration was 2.8 ng $ml^{-1}$ or 3.2% of the injected dose of antibody. By 24 h, the mean concentration was 1.9 ng $ml^{-1}$ and by 48 h only 3 patients had detectable levels with a mean concentration of 1.3 ng ml.$^{-1}$

Excretion of radioactivity in the urine throughout each 24 h period was the same: 8.6% $\pm$10.6 (day 1), 8.4% $\pm$9.0 (day 2) and 7.5% $\pm$5.0 (day 3). Thus 72 h after labelled antibody infusion, 25% of the radioactivity had been excreted.

Excretion in the faeces, however, was minimal. After 24 h 0.09 $\pm$0.14 of the injected activity was excreted and in the second 24 h 0.13 $\pm$0.16 were excreted.

### Discussion

RIL is potentially a tumour specific method of investigation. At present because a tumour specific antigen has yet to be identified, the technique depends on quantitative differences in the expression of antigens, such as CEA, between tumour and normal tissues. In this study we have evaluated potential clinical roles for RIL in the management of gastric and colorectal cancer. Although the scans have been interpreted by one of the investigators with prior knowledge of the extent of disease, independent assessment was not considered of value as not only does the technique remain to be standardised but potential sources of error are yet to be eliminated.

The results of the scans are comparable with reported series. Most of the primary tumours and the majority of the established recurrences have been detected. However, the experience of this and other studies demonstrates that tumour size is a significant limiting factor. Although some of the nodal secondary deposits were identified, scanning resolution was insufficient to allow reliable detection of small tumour volumes for accurate preoperative staging or for the detection of early recurrent disease. This limit of resolution, however, is no worse than for CT as the comparative study demonstrates similar numbers of false negative scans for either modality reflecting the failure to detect small lesions.

The measurement of radioactivity within tumours has indicated that both primary and secondary lesions accumulate more labelled antibody than surrounding normal tissues. The amount accumulated, however, is so low that detectable contrast from normal tissues cannot be reliably achieved by external scanning. Attempts to increase the tumour to normal tissue contrast have evaluated different isotopes (Fairweather *et al.*, 1983*b*; Rainsbury, 1984) and different methods of scanning such as emission tomography (Berche *et al.*, 1982). These techniques, however, are methods of improving the sensitivity of the detection of the very small amounts of labelled antibody accumulated by tumours. In order for tumour localisation to be effective for both diagnostic imaging and for drug targetting, larger proportions of injected antibody need to be accumulated. Preliminary studies have demonstrated that combinations of antibodies to different antigens increase the rate of detection of tumours presumably reflecting increased uptake of the injected preparations (Chatal *et al.*, 1984). Alternatively, increased amounts of antibody to the same antigen may produce similar results. The presently available antibodies are not ideal and it remains to be seen whether more appropriate antibodies either in combination or in larger doses will improve the results of tumour localisation.

The pharmacokinetic studies indicate potentially significant difficulties for the use of mouse monoclonal antibodies. Previous studies of labelled autologous gamma globulin have demonstrated a biphasic clearance over a period of fourteen days (Myant, 1952). The results of this study for the vascular distribution of radioactivity are in concurrence with these studies. However the rapid clearance of antibody suggests that radioactivity changes are not a reflection of changes in antibody levels. The reasons for this apparent discrepancy are not clear but several factors may be involved. Firstly detachment of $^{131}I$ from the antibody may occur. Approximately 25% of the injected radioactivity was excreted via the kidneys in the first 3 days which since all patients had apparently normal renal function must represent free iodine.

Secondly the mouse monoclonal antibody may evoke a response in the recipient possibly through the immune system. There is already evidence that patients can develop antimouse antibodies (Pimm *et al.*, 1985*b*) and these may inactivate or modify the administered antibody. Alternatively antibody may become non specifically attached to circulating cellular components. Whatever the mechanism such modification would not only influence reactivity with tumour bound CEA but also would affect the reaction in the ELISA used to estimate antibody concentration. Thus the ELISA may not represent an accurate assessment of the serum or tissue antibody levels. Further work is in progress to evaluate any modifications in the antibody and alternative methods of determining antibody concentrations.

The technique of tumour localisation by labelled antibodies has great potential in the assessment of malignant disease. In this study uptake by primary and secondary deposits has been demonstrated. However, the absolute amount of labelled preparation accumulated is insufficient to allow accurate tumour detection and thus limits the widespread use of the technique in clinical practice. Further work with particular reference to the pharmacokinetics of labelled antibodies is required to determine whether these difficulties can be overcome before the technique can be evaluated in independent clinical trials.

The help and advice of Mr S. Chandler, Mrs Z. Drolc and other members of the Department of Nuclear Medicine is gratefully acknowledged.

Thanks also to Mr V. Trend, Department of Bacteriology for carrying out the sterility testing of labelled antibody.

### References

BERCHE, C., MACH, J.P., LUMBROSO, J.D. & 7 others. (1982). Tomoscintigraphy for detecting gastrointestinal and medullary thyroid cancers: First clinical results using radiolabelled monoclonal antibodies against CEA. *Br. Med. J.*, **285**, 1447.

BRADWELL, A.R., DYKES, P.W. & FAIRWEATHER, D.J. (1983). Perchlorate blocking for radioimmunodetection. *J. Nucl. Med.*, **24**, 1081.

CHATAL, J.F., SACCAVINI, J.C., FUMOLEAU, P. & 5 others. (1984). Immunoscintigraphy of colon carcinoma. *J. Nucl. Med.*, **25**, 307.

CROWSON, M.C., HOCKEY, M.S., NEWMAN, J., STOKES, H.J., MACDONALD, F. & FIELDING, J.W.L. (1984). An immunocytochemical study of CEA expression in colorectal tumours and their metastases. *Br. J. Surg.* **71**, 376.

FAIRWEATHER, D.S., IRWIN, M., BRADWELL, A.R., DYKES, P.W. & FLINN, R.M. (1983*a*). Computer analysis of antibody scans. *Prot. Biol. Fluids*, **31**, 285.

FAIRWEATHER, D.S., BRADWELL, A.R., DYKES, P.W., VAUGHAN, A.T., WATSON-JAMES, S.F. & CHANDLER, S. (1983*b*). Improved tumour localisation using Indium 111 labelled antibodies. *Br. Med. J.*, **287**, 167.

GOLDENBERG, D.M., KIM, E.E., DELAND, F.H., BENNETT, S. & PRIMUS, F.J. (1980). Radioimmunodetection of cancer with radioactive antibodies to carcinoembryonic antigen. *Cancer Res.*, **40**, 2984.

HEYDERMAN, E. & NEVILLE, A.M. (1977). A shorter immunoperoxidase technique for the demonstration of carcinoembryonic antigen and other cell products. *J. Clin. Path.*, **30**, 138.

HOCKEY, M.S., STOKES, H.J., THOMPSON, H., WOODHOUSE, C.S., MACDONALD, F., FIELDING, J.W.L. & FORD, C.H.J. (1984). Carcinoembryonic antigen (CEA) expression and heterogeneity in primary and autologous metastatic gastric tumours demonstrated by a monoclonal antibody. *Br. J. Cancer*, **49**, 129.

HUNTER, W.M. & GREENWOOD, F.C. (1962). Preparation of Iodine-131 labelled human growth hormone of high specific activity. *Nature*, **194**, 495.

KOHLER, G. & MILSTEIN, C. (1975). Continuous culture of fused cells secreting antibody of predefined specificity. *Nature*, **256**, 495.

MACH, J.P., CARREL, S., FORNI, M., RITSHARD, J., DONATH, A. & ALBERTO, P. (1980). Tumour localisation of radiolabelled antibodies against carcinoembryonic antigen in patients with carcinoma. *New Eng. J. Med.*, **303**, 5.

MACH, J.P., BUCHEGGER, F., FORNI, M. & 7 others. (1981). Use of radiolabelled monoclonal anti-CEA antibodies for the detection of human carcinomas by external photoscanning and tomoscintigraphy. *Immunology Today*, **2**, 239.

MYANT, N.B. (1952). Observations on the metabolism of human gamma globulin labelled by radioactive iodine. *Clin. Sci.*, **11**, 191.

PIMM, M.V., ARMITAGE, N.C., PERKINS, A.C., SMITH, W. & BALDWIN, R.W. (1985*a*). Localisation of an antiCEA monoclonal antibody in colorectal carcinoma xenografts. *Cancer Immunol. Immunother.*, **19**, 8.

PIMM, M.V., ROWE, R., PERKINS, A.C. & BALDWIN, R.W. (1985*b*). Development of antimouse IgG and anti-idiotypic antibodies by patients receiving radiolabelled monoclonal antibody (791T/36) for diagnostic immunoscintigraphy. *Br. J. Cancer*. (In Press).

RAINSBURY, R.M. (1984). The localisation of human breast carcinomas by radiolabelled monoclonal antibodies. *Br. J. Surg.*, **71**, 805.

WOODHOUSE, C.S. (1982). An investigation of human lung tumour antigens. Ph.D. Thesis, University of Birmingham.

WOODHOUSE, C.S., FORD, C.H.J. & NEWMAN, C.E. (1981). A semi-automated enzyme-linked immunosorbent assay (ELISA) to screen for hybridoma cultures producing antibody to carcinoembryonic antigen. *Prot. Biol. Fluids*, **29**, 641.